# Non-pseudomonal Ecthyma Gangrenosum and Idiopathic Myelofibrosis in a Two-Year-Old Girl

**DOI:** 10.7759/cureus.2441

**Published:** 2018-04-06

**Authors:** Muniba Firoz, Ammarah Jamal, Syed Inam Ur Rehman

**Affiliations:** 1 Department of Pediatrics, Civil Hospital Karachi, Dow University of Health Sciences (DUHS), Karachi, PAK; 2 Department of Pediatrics,, Civil Hospital Karachi, Dow University of Health Sciences (DUHS), Karachi, PAK; 3 Department of Internal Medicine, Civil Hospital Karachi, Dow University of Health Sciences (DUHS), Karachi, PAK

**Keywords:** ecthyma, myelofibrosis, ecthyma gangrenosum, escherichia coli (e. coli)

## Abstract

Ecthyma gangrenosum is a skin lesion consequent to bacteremia, mostly due to Pseudomonas aeruginosa, although it may develop secondary to other organisms as well. The disease is often witnessed in patients with leukemia; however, a few cases of ecthyma gangrenosum in adults were reported to be associated with myelofibrosis. We report a case of ecthyma gangrenosum due to Escherichia coli (E. coli) in a two-year-old girl with idiopathic myelofibrosis.

## Introduction

Ecthyma gangrenosum (EG) is a skin lesion occurring in presence of bacteremia, mostly due to Pseudomonas aeruginosa. There is ample evidence that it may occur secondary to other organisms as well, even occurring in the absence of bacteremia [1].  Immunocompromised children, such as those with leukemia, are often affected by it; in adults, the condition has also been linked to myelofibrosis [2-3]. Myelofibrosis, a disorder of hematopoietic stem cells, seen rarely in the pediatric population, is characterized by poor proliferation of one or more: myeloid lineage markers, reactive fibrosis of the bone, and extramedullary hematopoiesis. We present a two-year-old girl with a rare case of EG due to Escherichia coli (E. coli) and idiopathic myelofibrosis.

## Case presentation

**Figure 1:**
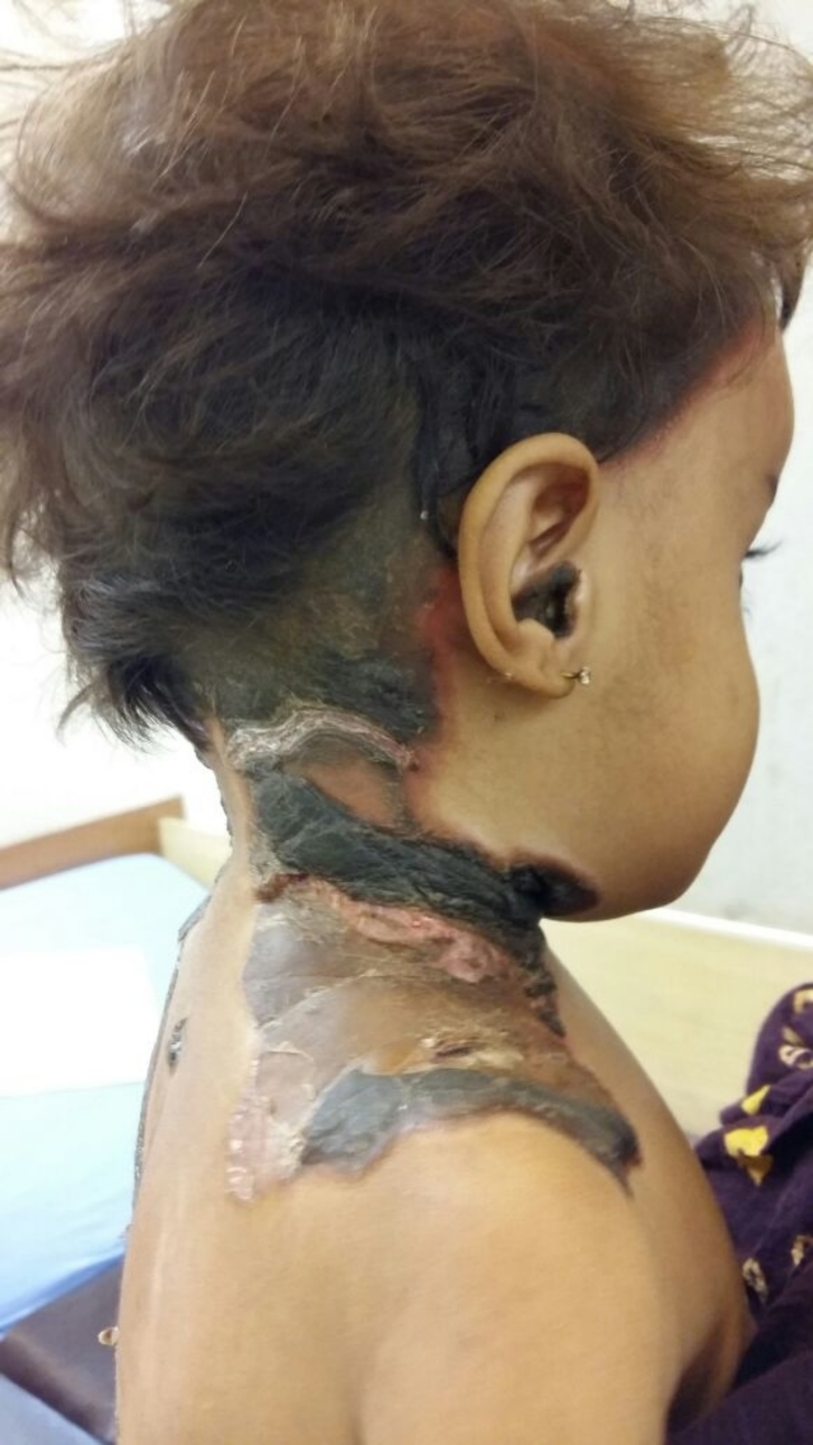
Black scabs on scalp extending down towards the neck and in the external ear

**Figure 2:**
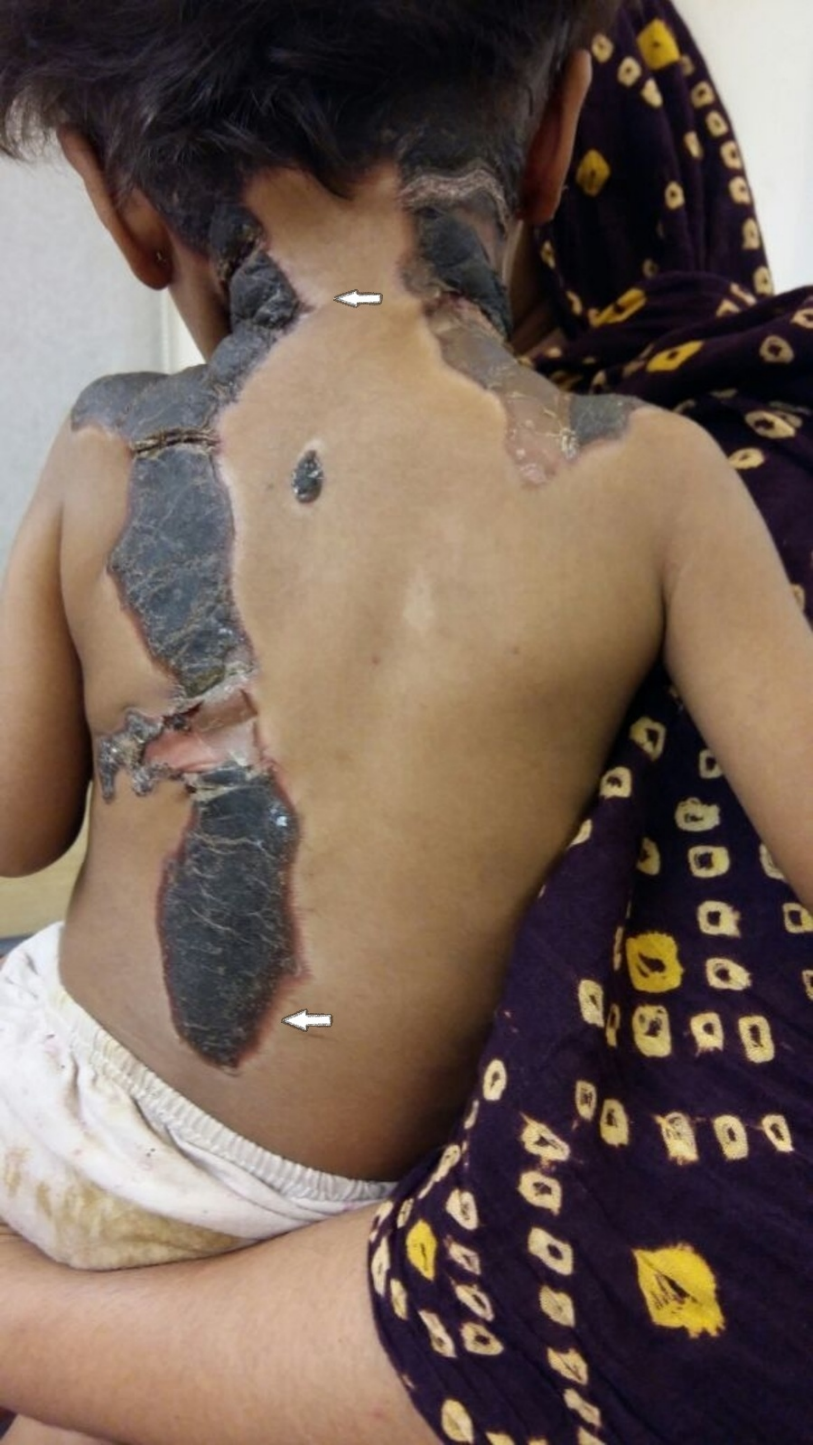
Large black irregular scabs with peripheral hypopigmentation and redness at different areas

A two-year-old girl was admitted to the pediatric ward with a 15-day history of progressive skin lesions. She had another hospitalization six months earlier due to severe anemia and thrombocytopenia secondary to malaria and received packed cells and platelet transfusions at that time. She was unvaccinated and malnourished, but developmentally appropriate, with an unremarkable family history. Clinically, she looked pale, having large, black, irregular, and thick scabs over her scalp, neck, external ear, back, perineum, and groin. The scabs were surrounded by hypopigmentation in some areas and redness in others but had no discharge and were not itchy (Figures 1, 2).The only positive findings on the rest of her examination were an enlarged liver and spleen measuring 4 cm and 3 cm, respectively, below the costal margins.

Initial differential diagnosis for the skin lesions was EG and necrotizing fasciitis. Tuberculosis and leishmaniasis were considered as differentials since they can present with similar skin lesions and hepatosplenomegaly. Possibilities of acute leukemia and megaloblastic anemia with a severe skin infection were also taken into account because of hepatosplenomegaly. Upon investigation, a full blood count showed moderate anemia and severe thrombocytopenia with a hemoglobin of 7.9 g/dL, platelet count of 12 x 10^9^/L, respectively, and a normal white blood cell count of 11 x 10^9^/L, with 57% neutrophils and 41% lymphocytes. Mean corpuscular volume (MCV) was 80.2 fL. Peripheral blood film revealed no circulating blast cells. Chest x-ray was normal, while an ultrasound of the abdomen confirmed the clinical finding of hepatosplenomegaly. Investigations for tuberculosis turned out to be negative. A serum vitamin B12 level was low, but serum iron and ferritin levels were within normal limits.

The child was started on empirical broad-spectrum antibiotic coverage using intravenous ampicillin and gentamicin, along with soaking the lesions with normal saline. Over the next few days, pus started to ooze from the lesions and grew Escherichia coli (E. coli) on culture medium. There was no growth on blood cultures. The antibiotics were changed to ceftazidime in accordance with the culture and sensitivity report with consequent rapid improvement. Additional treatment included packed cell and platelet transfusions, vitamin B12 replacement, vaccination, and nutritional rehabilitation. At the one month follow-up, the child had recovered otherwise, except for persistent bicytopenia. Therefore, we proceeded with a bone marrow biopsy, which revealed cellular marrow, prominent erythropoiesis, and Grade II reticulin fibrosis, findings consistent with the diagnosis of myelofibrosis. However, there was no evidence of hemoparasite (malaria, Leishmania), hemophagocytosis, granuloma, storage cells, blast cells, overt dysplasia, or malignancy. Diseases that could lead to secondary myelofibrosis in children were ruled out in our patient via bone marrow examination (tuberculosis, leishmaniasis, Gaucher disease), negative clinical features (such as juvenile idiopathic arthritis), and laboratory investigations (Table 1). Our final diagnosis was EG secondary to E. coli with idiopathic myelofibrosis. The child has been under the care of a pediatric hematologist for the past two years, receiving supportive therapy. There has been no improvement or worsening of her condition.

**Table 1:** Investigations for Causes of Secondary Myelofibrosis HPLC: high-performance liquid chromatography: ANA: antinuclear antibody;  Anti-DSn DNA: anti-double stranded deoxyribonucleic acid

CAUSES OF SECONDARY MYELOFIBROSIS	INVESTIGATIONS
Langerhans cell histiocytosis	Normal Skeletal survey
Osteopetrosis	
Hemophagocytic lymphohistiocytosis	Ferritin: 711 ng/ml, fibrinogen: 390 mg/dl
Sickle cell anemia	HPLC normal
Fanconi anemia	Normal chromosomal breakage study
Vitamin D deficiency	Vitamin D level: 24.2 mg/dl
Systemic lupus erythematosus	ANA and Anti-DS DNA - negative
Gray platelet syndrome	No megakaryocytes
Hyperparathyroidism	Normal calcium and phosphorous
Renal osteodystrophy	Normal renal profile

## Discussion

Ecthyma gangrenosum (EG) is a rare, yet well-characterized, dermatological manifestation of a severe infection usually from Pseudomonas aeruginosa. Other organisms, such as E. coli and Staphylococcus aureus, have also been implicated and rarely EG has been reported with herpes simplex, too. Clinically, it is described as red macules which progress into papules, turning into hemorrhagic bullae, and eventually ulcerate with black scab formation over the lesion. Immunosuppression, neutropenia, and bacteremia are noted clinical observations, which have a high mortality associated with it [4].

In light of the aforementioned clinical parameters, our case is highly unique in three aspects. Firstly, in our patient, the causative organism for EG was Escherichia coli, which has been discussed in only nine case reports before us [5].

The second unique aspect was the absence of neutropenia in our case despite having bone marrow suppression and myelofibrosis, a finding comparable to a report by DeLario et al., who demonstrated neutropenia in only seven out of 19 cases of myelofibrosis [6].

The third unique feature of our case was the development of EG in the absence of neutropenia. We were able to find in the literature that myelofibrosis is associated with an increased susceptibility to infections, even in absence of leukopenia/neutropenia [7]. Therefore, we can safely presume that the underlying myelofibrosis in our patient was responsible for the development of her severe skin infection.

Treatment of EG involves the use of appropriate antibiotics according to the underlying etiologic pathogen and surgical debridement of necrotic tissue if needed. Bacteremia and delayed diagnosis are factors for a poor prognosis [8]. Our patient also showed a good response to appropriate antibiotics. In a similar case study of an adult patient with myelofibrosis and persistent lesions of EG for about four weeks, granulocyte-macrophage colony stimulating factor (GM-CSF) was successfully used in the treatment regimen [3]. This indicates that supportive therapies to correct cytopenias can also be used for better outcomes.

The common mutations of JAK2V617F or MPLW515 K/L in adult patients with myelofibrosis are not reported in pediatric cases [9]. According to a recent study of Chinese pediatric patients with idiopathic myelofibrosis, calreticulin (CALR) mutations were detected in 50% of the cases, suggesting that CALR mutation screening could be used as a molecular marker for the diagnosis of pediatric patients with idiopathic myelofibrosis [10]. However, due to the lack of genetic analysis facilities in our setup, the diagnosis must rely heavily on clinical and laboratory factors and the exclusion of secondary disorders to make an accurate diagnosis of idiopathic myelofibrosis. Myelofibrosis in children is less frequently associated with malignancies. It progresses uneventfully with supportive treatment and even remits on rare occasions, but some children succumb to overwhelming infections or complications of therapy. The only curative option for myelofibrosis is stem cell transplantation; other treatment modalities, such as androgens and progesterone, are reported in the literature with little success.

## Conclusions

We conclude that skin lesions may be a manifestation of serious underlying systemic disease and, therefore, should always be investigated in detail to avoid unnecessary delays in their diagnosis and treatment. By reporting this case, we intend to share this unique presentation of idiopathic myelofibrosis with EG and thus add to the pool of these cases for a better understanding of these conditions.
